# H_2_Mab-19, an anti-human epidermal growth factor receptor 2 monoclonal antibody exerts antitumor activity in mouse oral cancer xenografts

**DOI:** 10.3892/etm.2020.8765

**Published:** 2020-05-18

**Authors:** Junko Takei, Mika Kato Kaneko, Tomokazu Ohishi, Manabu Kawada, Hiroyuki Harada, Yukinari Kato

**Affiliations:** 1 Department of Antibody Drug Development, Tohoku University Graduate School of Medicine, Sendai, Miyagi 980-8575, Japan; 2 Department of Oral and Maxillofacial Surgery, Graduate School of Medical and Dental Sciences, Tokyo Medical and Dental University, Tokyo 113-8510, Japan; 3 Institute of Microbial Chemistry (BIKAKEN), Numazu, Microbial Chemistry Research Foundation, Numazu-shi, Shizuoka 410-0301, Japan; 4 New Industry Creation Hatchery Center, Tohoku University, Sendai, Miyagi 980-8575, Japan

**Keywords:** HSC-2, monoclonal antibody, antitumor activity

## Abstract

Human epidermal growth factor receptor 2 (HER2) is reported to be overexpressed in breast cancers and is associated with poor clinical outcome. Trastuzumab is a humanized anti-HER2 antibody that offers significant survival benefits to patients with HER2-overexpressing breast cancer. In this study, a novel anti-HER2 monoclonal antibody (mAb), H_2_Mab-19 (IgG_2b_, kappa) was developed. Antibody-dependent cellular cytotoxicity (ADCC), complement-dependent cytotoxicity (CDC), and antitumor activity of H_2_Mab-19 were investigated using both breast cancer and oral cancer cell lines. H_2_Mab-19 demonstrated cytotoxicity in BT-474 (a human breast cancer cell line) and HSC-2 or SAS (human oral cancer cell lines). H_2_Mab-19 also possessed both ADCC and CDC activity against BT-474, HSC-2, and SAS cell lines. In comparison to control mouse IgG, H_2_Mab-19 significantly reduced tumor development in BT-474, HSC-2, and SAS xenografts. Collectively, these results suggest that treatment with H_2_Mab-19 may be a useful therapy for patients with HER2-expressing breast and oral cancers.

## Introduction

The overexpression of human epidermal growth factor receptor 2 (HER2) is reported in breast (1,2), gastric (3), pancreatic (4), lung (5), and colorectal cancers (6). This expression is associated with poor clinical outcomes in patients with HER2-positive breast cancer (1,2). Humanized anti-HER2 monoclonal antibodies (mAbs) trastuzumab and pertuzumab have been used in the treatment of HER2-positive breast cancer (7-9). Treatment with trastuzumab resulted in significant survival benefits these patients (10). In comparison to trastuzumab monotherapy, the combination of trastuzumab and pertuzumab with chemotherapy has led to significant improvements in overall survival (11).

Trastuzumab deruxtecan (DS-8201), a recently developed drug, is comprised of three components, a novel enzyme-cleavable linker, and a topoisomerase I inhibitor (12). Even in low-HER2-expressing tumors, DS-8201 shows antitumor activity. This drug has several innovative features: i) a highly potent, novel payload with a high drug-to-antibody ratio, ii) good homogeneity, iii) a tumor-selective cleavable linker, iv) a stable linker-payload in circulation, and v) a cytotoxic agent with a short *in vivo* half-life *in vivo* (13). Furthermore, the cytotoxic payload can exert a bystander effect (13).

The novel anti-HER2 mAb (H_2_Mab-19) developed in this study was investigated for its antitumor activities in mouse xenograft models of breast and oral cancers. These properties have not been previously investigated with regard to HER2 expression.

## Materials and methods

### 

#### Cell lines

Oral squamous carcinoma cell lines including Ca9-22 (derived from gingiva), HO-1-u-1 (mouth floor), HSC-2 (oral cavity), and SAS (tongue) were obtained from the Japanese Collection of Research Bioresources Cell Bank (Osaka, Japan). LN229 (glioblastoma cell line), MDA-MB-468 (breast cancer), BT-474 (breast cancer), and P3U1 (mouse myeloma) were obtained from the American Type Culture Collection. LN229/HER2 cells were established in a previous study (14). P3U1 cells were cultured in RPMI-1640 medium (Nacalai Tesque, Inc., Kyoto, Japan). LN229, LN229/HER2, MDA-MB-468, BT-474, Ca9-22, HO-1-u-1, HSC-2, and SAS were cultured in Dulbecco's modified Eagle's medium (DMEM; Nacalai Tesque, Inc.) supplemented with 10% heat-inactivated fetal bovine serum (Thermo Fisher Scientific Inc.), 100 units/ml of penicillin, 100 µg/ml streptomycin, and 25 µg/ml amphotericin B (Nacalai Tesque, Inc.) at 37°C in a humidified atmosphere containing 5% CO_2_.

#### Animals

All animal experiments were performed in accordance with relevant guidelines and regulations to minimize animal suffering and distress in the laboratory. Animal experiments for hybridoma production were approved by the Animal Care and Use Committee of Tohoku University (permit no. 2016MdA-153). Animal health was monitored daily. Animal studies for Antibody-Dependent Cellular Cytotoxicity were approved by the institutional committee for experiments of the Institute of Microbial Chemistry (permit no. 2019-066). Animal studies for antitumor activity were approved by the institutional committee for experiments of the Institute of Microbial Chemistry (permit no. 2019-014). Mice were monitored for health and weight every 3 or 4 days. Experiment duration was three weeks. A bodyweight loss exceeding 25% and a maximum tumor size exceeding 3,000 mm^3^ were identified as humane endpoints. Mice were euthanized by cervical dislocation, and the death was verified by respiratory arrest and cardiac arrest.

#### Hybridoma production

One four-week-old female BALB/c mouse was purchased from CLEA Japan and housed under specific pathogen-free conditions. Anti-HER2 hybridoma cells were produced as described previously (14). Briefly, the BALB/c animal was immunized by intraperitoneal (i.p.) administration of 100 µg recombinant HER2 extracellular domain along with Imject Alum (Thermo Fisher Scientific Inc.). After several additional immunizations, a booster dose was administered i.p. 2 days before harvesting spleen cells. Mice were euthanized by cervical dislocation, and the death was verified by respiratory arrest and cardiac arrest. Spleen cells were then fused with P3U1 cells using PEG1500 (Roche Diagnostics, Indianapolis, IN, USA). The resulting hybridoma cells were grown in RPMI medium supplemented with hypoxanthine, aminopterin, and thymidine selection medium (Thermo Fisher Scientific, Inc.). Culture supernatants were screened using enzyme-linked immunosorbent assays with recombinant HER2 extracellular domain. mAbs were purified from the supernatants of hybridoma cells and cultured in Hybridoma-SFM medium (Thermo Fisher Scientific, Inc.) using Protein G Sepharose 4 Fast Flow (GE Healthcare UK Ltd.).

#### Flow cytometry

Hybridoma cells were harvested by brief exposure to 0.25% trypsin/1-mM ethylenediaminetetraacetic acid (EDTA; Nacalai Tesque, Inc.). After washing with 0.1% bovine serum albumin in phosphate-buffered saline (PBS), cells were treated with 1 µg/ml anti-HER2 (H_2_Mab-19) for 30 min at 4°C and subsequently with Alexa Fluor 488-conjugated anti-mouse IgG (1:1,000; Cell Signaling Technology, Inc.). Fluorescence microscopy data were collected using an EC800 Cell Analyzer (Sony Corp.).

#### Immunohistochemical analyses for formalin-fixed paraffin-embedded (FFPE) tissues

Histologic sections (catalog no. T8235721-5; lot no. B104066; BioChain Institute Inc.) were purchased in this study. Four-µm histologic sections from paraffin blocks of resected xenografts were also produced. These sections were deparaffinized in xylene, then rehydrated and autoclaved in citrate buffer (pH 6.0; Agilent Technologies Inc.) for 20 min. Sections were incubated with primary mAbs for 1 h at room temperature, then treated using an Envision+ kit (Agilent Technologies Inc.) for 30 min. Color was developed using 3,3-diaminobenzidine tetrahydrochloride (Agilent Technologies Inc.) for 2 min, and sections were then counterstained with hematoxylin (FUJIFILM Wako Pure Chemical Corporation).

#### Immunohistochemical analyses for frozen tissues

Histologic sections (catalog no. T6235086-1, BioChain Institute Inc.) were incubated with 1 µg/ml of primary mAbs for 1 h at room temperature and were then treated using an Envision+ kit (Agilent Technologies Inc.) for 30 min. Color was developed using 3,3-diaminobenzidine tetrahydrochloride (Agilent Technologies Inc.) for 2 min, and sections were then counterstained with hematoxylin (FUJIFILM Wako Pure Chemical Corporation).

#### Determination of the binding affinity

Cells were suspended in 100 µl serially diluted H_2_Mab-19 (6 ng/ml-100 µg/ml), followed by the addition of Alexa Fluor 488-conjugated anti-mouse IgG (1:200; Cell Signaling Technology, Inc.). Fluorescence microscopy data were collected using an EC800 Cell Analyzer (Sony Corp.). The dissociation constant (*K*_D_) was obtained by fitting binding isotherms to built-in one-site binding models in GraphPad PRISM 6 (GraphPad Software, Inc.).

#### Antibody-dependent cellular cytotoxicity

Six six-week-old female BALB/c nude mice were purchased from Charles River. After euthanization by cervical dislocation, spleens were removed aseptically and single-cell suspensions obtained by forcing spleen tissues through a stainless steel mesh using a syringe. Erythrocytes were lysed with a 10-sec exposure to ice-cold distilled water. Splenocytes were washed with DMEM and resuspended in DMEM with 10% FBS and used as effector cells. Target cells were labeled with 10-µg/ml Calcein AM (Thermo Fisher Scientific, Inc.) and resuspended in the same medium. The target cells (2x10^4^ cells/well) were plated in 96-well plates and mixed with effector cells, anti-HER2 antibodies, or control IgG (mouse IgG_2b_) (Sigma-Aldrich Corp.). After a 4-h incubation, the Calcein AM release of supernatant from each well was measured. Fluorescence intensity was determined using a microplate reader (Power Scan HT) (BioTek Instruments) with an excitation wavelength of 485 nm and an emission wavelength of 538 nm. Cytolytic activity (as % of lysis) was calculated as: % lysis=(E-S)/(M-S) x100, where E is fluorescence of combined target and effector cells, S is spontaneous fluorescence of target cells only, and M is maximum fluorescence measured after lysing all cells with a buffer containing 0.5% Triton X-100, 10 mM Tris-HCl (pH 7.4), and 10 mM of EDTA.

#### Complement-dependent cytotoxicity

Cells in DMEM supplemented with 10% FBS (2x10^4^ cells/well) were plated in 96-well plates., and incubated for 5 h at 37°C with either anti-HER2 antibodies or control IgG (mouse IgG_2b_) (Sigma-Aldrich Corp.) and 10% of rabbit complement (Low-Tox-M Rabbit Complement) (Cedarlane Laboratories). To assess cell viability, an MTS [3-(4,5-dimethylthiazol-2-yl)-5-(3-carboxymethoxyphenyl)-2-(4-sulfophenyl)-2H-tetrazolium; inner salt] assay was performed using a CellTiter 96 AQueous assay kit (Promega).

#### Antitumor activity of H_2_Mab-19 in the xenografts of breast cancers

Sixteen six-week-old female BALB/c nude mice were purchased from Charles River (Kanagawa, Japan) and used at 10 weeks of age. BT-474 cells (0.3 ml of 1.33x10^8^ cells/ml in DMEM) were mixed with 0.5 ml BD Matrigel Matrix Growth Factor Reduced (BD Biosciences). One hundred-µl of this suspension (5x10^6^ cells) was injected subcutaneously into the left flank. After day 1, 100 µg H_2_Mab-19 and control mouse IgG (Sigma-Aldrich Corp.) in 100 µl PBS were injected i.p. into treated and control mice, respectively. Additional antibodies were then injected on days 7 and 14. Eighteen days after cell implantation, all mice were euthanized by cervical dislocation and tumor diameters and volumes were determined as previously described (15).

#### Antitumor activity of H_2_Mab-19 in xenografts of oral cancers

Thirty-two six-week-old female BALB/c nude mice were purchased from Charles River and used at 10 weeks of age. HSC-2 or SAS cells in DMEM (0.3 ml with 1.33x10^8^ cells/ml) were mixed with 0.5 ml BD Matrigel Matrix Growth Factor Reduced (BD Biosciences). A 100-µl suspension containing 5x10^6^ cells was injected subcutaneously into the left flank. After day 1, 100 µg H_2_Mab-19 and control mouse IgG (Sigma-Aldrich Corp.) in 100 µl PBS were injected i.p. into treated and control mice, respectively. Additional antibodies were then injected on days 6 and 14. Twenty days after cell implantation, all mice were euthanized by cervical dislocation. Tumor diameters and volumes were determined as previously described (15).

#### Statistical analyses

All data were expressed as mean ± SEM. Statistical analysis used ANOVA and Tukey-Kramer's test with GraphPad Prism 6 (GraphPad Software, Inc.). *P<0.05 was considered to indicate a statistically significant difference.*

## Results

### 

#### Production of anti-HER2 mAb

**Figure 1:**
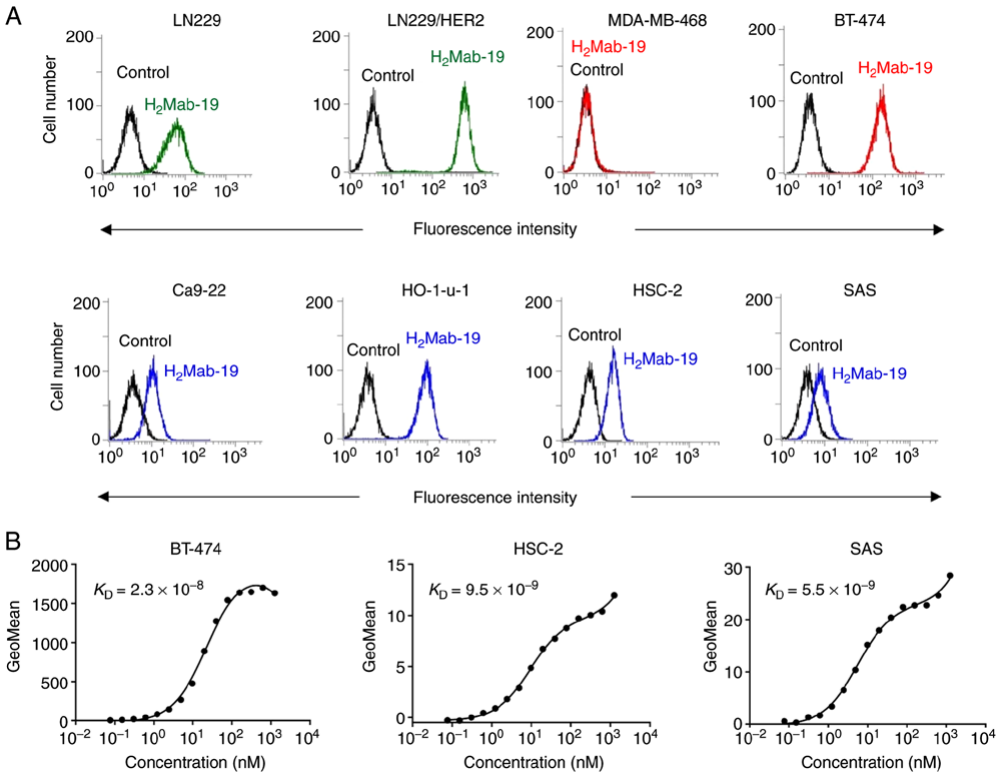
Characterization of H_2_Mab-19 using flow cytometry. (A) Glioblastoma cell lines (LN229, LN229/HER2), breast cancer cell lines (MDA-MB-468, BT-474), and oral cancer cell lines (Ca9-22, HO-1-u-1, HSC-2, SAS) were treated with H_2_Mab-19. Black line is negative control (PBS). (B) Determination of binding affinity of H_2_Mab-19 for BT-474, HSC-2, and SAS using flow cytometry.

One mouse was immunized with the recombinant extracellular domain of HER2(16), purified using the MAP tag system (17). Flow cytometry was performed to check reactions with the LN229 cells (glioblastoma) and HER2-overexpressing LN229 cells (LN229/HER2). LN229 cells endogenously express HER2 and some reaction with these cells was expected. The overexpression of HER2 in LN229/HERS2 cells would produce a stronger reaction. One IgG_2b_ subclass clone of H_2_Mab-19 was obtained, though almost all mAbs were in the mouse IgG_1_ subclass. H_2_Mab-19 reacted with LN229/HER2 and weakly reacted with LN229 cells (Fig. 1A), indicating that H_2_Mab-19 is specific to HER2.

#### Characterization of H_2_Mab-19

H_2_Mab-19 recognized endogenous HER2 in a breast cancer cell line, BT-474, which is HER2-positive (18), but did not react with a breast cancer cell line, MDA-MB-468, which is HER2-negative (18) (Fig. 1A). Further, H_2_Mab-19 strongly reacted with endogenous HER2 in HO-1-u-1 cells (oral cancer) and only weakly reacted with other oral cancer cell lines, Ca9-22, HSC-2, and SAS (Fig. 1A). Using flow cytometry, binding affinities (*K*_D_) of H_2_Mab-19 to BT-474, HSC-2, and SAS cell lines were 2.3x10^-8^, 9.5x10^-9^ and 5.5x10^-9^ M, respectively. These results indicate that H_2_Mab-19 maintains high affinity across HER2-expressing cell lines. H_2_Mab-19 did not stain FFPE-breast cancer tissues (Fig. S1). In contrast, H_2_Mab-19 reacted with frozen breast cancer tissues although the sensitivity of H_2_Mab-77 was better than that of H_2_Mab-19 (Fig. S2).

#### ADCC and CDC activities against breast and oral squamous cell carcinoma cell lines

**Figure 2:**
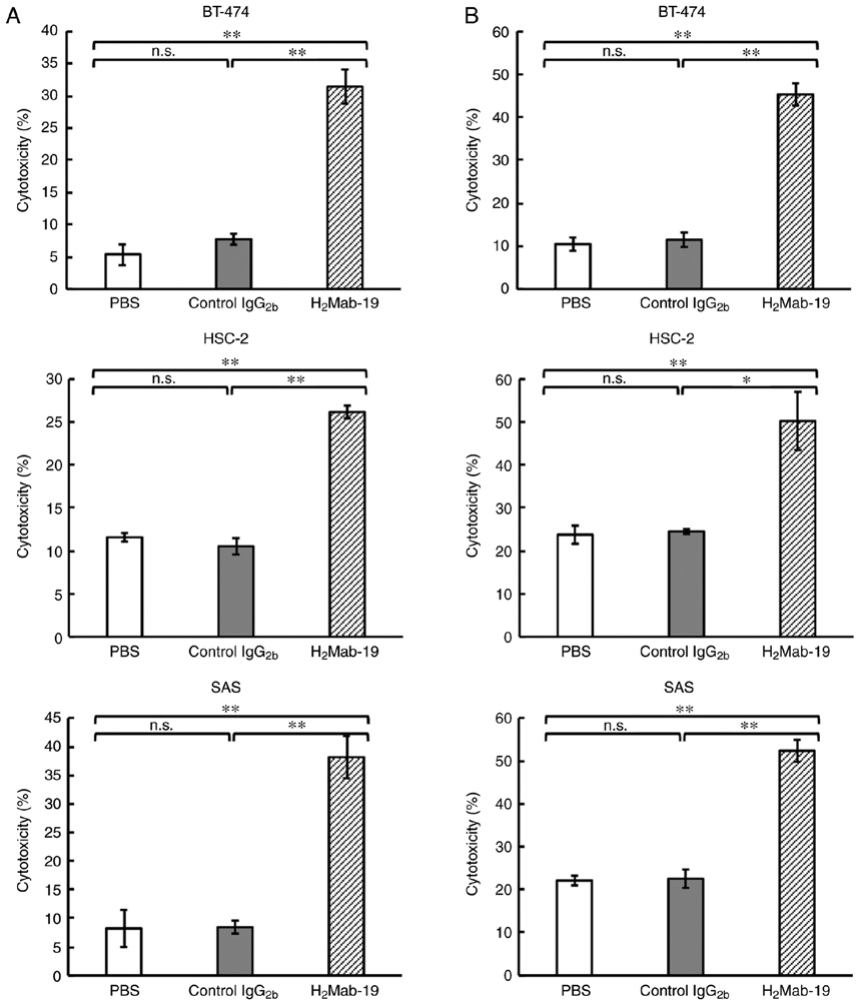
ADCC and CDC activity. (A) ADCC activity against BT474, HSC-2 and SAS cells. (B) CDC activity against BT474, HSC-2 and SAS cells. ^*^^*^P<0.01; ^*^P<0.05; n.s., not significant; ADCC, antibody-dependent cellular cytotoxicity; CDC, complement-dependent cytotoxicity.

This study examined whether H_2_Mab-19 induced ADCC and CDC in HER2-expressing breast or OSCC cell lines. H_2_Mab-19 was a mouse IgG_2b_ subclass antibody that could possess both ADCC and CDC. H_2_Mab-19 exhibited high ADCC activity against BT-474, HSC-2, and SAS cells (Fig. 2A). High CDC activity was also observed in BT-474, HSC-2, and SAS cells (Fig. 2B), suggesting that H_2_Mab-19 might exert antitumor activity *in vivo*.

#### Antitumor activity of H_2_Mab-19 in mouse xenografts of breast cancers

**Figure 3:**
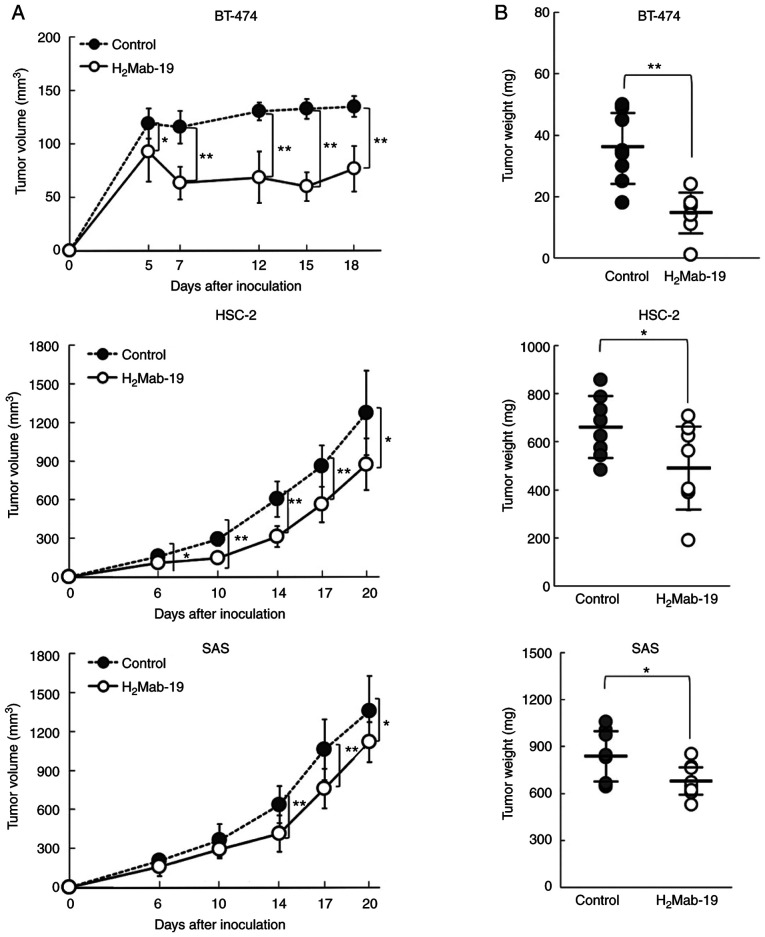
Evaluation of antitumor activity of H_2_Mab-19. (A) Tumor volume and (B) tumor weight measured from BT-474 (upper), HSC-2 (middle) and SAS (lower) xenografts. Values are mean ± SEM. ^*^^*^P<0.01; ^*^P<0.05.

To study the antitumor activity of H_2_Mab-19 on cell growth *in vivo*, BT-474 cells were implanted subcutaneously in the flanks of nude mice. H_2_Mab-19 and control mouse IgG were injected i.p. three times (days 1, 7, and 14 after cell injection) into treated and control mice, respectively. Tumor formation was observed in mice in both H_2_Mab-19-treated and control groups. H_2_Mab-19 treatment significantly reduced tumor development compared to development in control mice on days 5, 7, 12, 15, and 18 (Fig. 3A, upper). Weights of tumors from H_2_Mab-19-treated mice were significantly less than for tumors from IgG-treated control mice (Fig. 3B, upper). BT-474 xenografts on day 18 are shown in Fig. S3A. Resected tumors are depicted in Fig. S3B. Total body weight was not significantly different between the two groups (Fig. S3C). We could not show the histological data about the liver and kidney in this study. HER2 was highly expressed in all cancer cells of H_2_Mab-19-treated BT-474 and control xenografts (Fig. S4).

#### Antitumor activities of H_2_Mab-19 in the mouse xenografts of oral cancers

H_2_Mab-19 possessed antitumor activity in mouse xenografts of breast cancers. Whether this activity extended to xenografts of oral cancers was also assessed. BT-474 cells expressed high levels of HER2 (Fig. 1A); HER2 levels were however low in HSC-2 and SAS cells (Fig. 1B). Nevertheless, HSC-2 and SAS are useful for investigation of antitumor activity *in vivo* (16). Thus, HSC-2 and SAS were used for mouse xenografts of oral cancers.

Initially, HSC-2 cells were implanted subcutaneously into the flanks of nude mice. H_2_Mab-19 and mouse IgG were injected i.p. three times (on days 1, 6, and 14 after cell injections into treated and control mice, respectively. Tumor formation was observed in mice in both groups. In comparison to control mice, H_2_Mab-19-treated mice showed significantly reduced tumor development on days 6, 10, 14, 17 and 20 (Fig. 3A, middle). Weights of tumors from H_2_Mab-19-treated mice were significantly less than for tumors from control mice (Fig. 3B, middle). HSC-2 xenograft mice are shown on day 20 in Fig. S5A and resected tumors are depicted in Fig. S5B. Total body weights were not significantly different between the two groups (Fig. S5C). We could not show the histological data about the liver and kidney in this study. HER2 was not expressed in cancer cells of H_2_Mab-19-treated or control groups (Fig. S6). HER2 expression was diminished in HSC-2 xenografts, and H_2_Mab-19 did not exert effective antitumor activity.

For the second xenograft model of oral cancers, SAS cells were subcutaneously implanted into the flanks of nude mice. H_2_Mab-19 and mouse IgG were injected i.p. thrice, on days 1, 6, and 14 after cell injections into the mice, into treated and control mice, respectively. Tumor formation was observed in mice in both treated and control groups. In comparison to IgG-treated control mice, H_2_Mab-19 significantly reduced tumor development on days 14, 17, and 20 (Fig. 3A, lower). Weights of tumors from H_2_Mab-19-treated mice were significantly less than tumors from IgG-treated control Mice (Fig. 3B, lower). The SAS xenografts on day 20 are shown in Fig. S7A and resected tumors are depicted in Fig. S7B. Total body weights were not significantly different between the two groups (Fig. S7C). We could not show the histological data about the liver and kidney in this study. HER2 was not expressed in cancer cells of H_2_Mab-19-treated and control groups (Fig. S8). HER2 expression was diminished in SAS xenografts, and H_2_Mab-19 did not exert effective antitumor activity.

## Discussion

Using CasMab technology (19), several anti-HER2 mAbs, including H_2_Mab-77(14), H_2_Mab-119(20), and H_2_Mab-139(16) were identified. These antibodies are useful for flow cytometry, western blot, and immunohistochemical analyses. Because the subclass of these mAbs is mouse IgG_1_, they do not possess antibody-dependent cellular cytotoxicity (ADCC) or complement-dependent cytotoxicity (CDC).

The first objective of this study was the development of an anti-HER2 mAb in either IgG_2a_ or IgG_2b_ subclasses using CasMab technology. Both IgG_2a_ (21) and IgG_2b_ antibodies (22) show ADCC and CDC activity. The second objective was to investigate anti-HER2 activity using oral cancer cell lines; anti-HER2 mAbs have not been investigated for their activity against oral cancers. The first objective was met through isolation of H_2_Mab-19 from the IgG_2b_ subclass (Fig. 1). This antibody could then be used to investigate ADCC and CDC activity *in vitro* and antitumor activity *in vivo*. H_2_Mab-19 showed both ADCC and CDC activity against breast or oral cancer cell lines (Fig. 2). Further, H_2_Mab-19 exerted antitumor activity against both breast cancer and oral cancer xenografts (Fig. 3). These results demonstrated two important issues: i) anti-HER2 mAbs from the IgG_2b_ subclass could be developed using our original CasMab technology, and ii) anti-HER2 mAbs from IgG_2b_ subclass could possess ADCC, CDC, and antitumor activities. Recently, Fiedler *et al* reported that TrasGEX, an ADCC-enhanced version of trastuzumab, showed antitumor activity in 50% of evaluated patients from a phase I study (23). They showed that TrasGEX exhibited similar pharmacokinetics to those of trastuzumab and was safe and well-tolerated by patients with solid tumors. These data are consistent with the designation of HER2 as a promising target for the treatment of HER2-amplified tumors. Trastuzumab and TrasGEX are known as beneficial anti-HER2 mAbs for targeting breast or stomach cancers, H_2_Mab-19 could be also a useful tool for investigating ADCC, CDC, antitumor activities for oral cancers. Further investigation of the mechanism of antitumor activity by H_2_Mab-19, and the development of antibody-engineered antibodies, including chimeric or humanized H_2_Mab-19 or its single chain (sc) Fv, are aims for future studies.

Oral cancer accounts for approximately 2% of all cancer cases worldwide (24). Annually, more than 350,000 individuals are diagnosed with oral cancer and these diseases prove fatal for 170,000 of these people. Major risk factors for oral cancer are the use of tobacco and alcohol (25). Decreased smoking and drinking has resulted in a decline in the incidence of oral cancer. However, recent studies have reported an increase in the number of young patients diagnosed with these diseases (26,27).

More than 50% of oral cancers occur in tongue tissue and on the floor of the mouth. Other locations include the buccal mucosa, gingiva, lip and palate (28). HER2 expression was assessed in four oral cancer cell lines of different origin, including Ca9-22 (gingiva), HO-1-u-1 (mouth floor), HSC-2 (oral cavity), and SAS (tongue). HER2 expression was observed in all cell lines (Fig. 1), indicating that expression is independent of location in the oral cavity.

Oral cancers display several histological tumor types, including squamous cell carcinoma (SCC), adenocarcinoma, mucoepidermoid carcinoma, adeno cystic carcinoma and osteosarcoma. SCC is most common, accounting for over 90% of all disease (29). Treatment of oral SCC (OSCC) depends for the most part on stage. Early stages (stage-I and -II) are treated via surgery or radiotherapy (RT) alone. Advanced stages (stage-III and -IV) require a combination of surgery, RT and chemotherapy (CT) (30). Cisplatin (CDDP) is mainly used for CT of OSCCs, often combined with 5-fluorouracil (5-FU) and docetaxel (31,32). Other anticancer agents such as carboplatin, paclitaxel, and methotrexate (MTX) can be useful (33), but useful drugs with specific molecular targets are limited.

Cetuximab, a mouse-human chimeric antibody (IgG_1_) that targets epidermal growth factor receptor (EGFR), was recently approved for treatment of oral cancer. Several studies report its effectiveness against locoregionally advanced head and neck cancer and recurrent or metastatic squamous cell carcinoma of the head and neck (34-36). Advances in diagnosis and therapeutic techniques have improved the overall 5-year survival rate to 70%. However, the 5-year survival rate in stage IV is only 40% (37) and further treatments need to be developed. In this study, HER2 is shown to be expressed in oral cancers, and anti-HER2 mAbs have useful for antitumor activity. Thus, anti-HER2 therapies using trastuzumab could be valuable for oral cancer treatment. Immunohistochemically, HER2 expressed was reported in only 1.4% (38) of oral cancer, though it is expressed in 10.4% of breast cancers (39). Thus, targeting only HER2 may not be sufficient for treating oral cancers. Despite the low HER2 overexpression/amplification rate of only 1-2%, those few patients may possibly benefit from anti-HER2 therapy because an antitumor effect of combined gefitinib and trastuzumab or cetuximab and trastuzumab treatment on HNSCC *in vitro* were demonstrated (40,41). Pursuing multiple targets, such as EGFR and HER2, may be needed for effective therapy.

## Supplementary Material

Immunohistochemical analyses of paraffin sections of breast cancers using H_2_Mab-19 and H_2_Mab-77. Sections of breast cancers were incubated with H_2_Mab-19 (10 *μ*g/ml) or H_2_Mab-77 (1 *μ*g/ml) Sections were counterstained with hematoxylin. Scale bar=100 *μ*m.

Immunohistochemical analyses of frozen sections of breast cancers using H_2_Mab-19 or H_2_Mab-77. Sections of breast cancers were incubated with H_2_Mab-19 (1 *μ*g/ml) or H_2_Mab-77 (1 *μ*g/ml). Sections were then counterstained with hematoxylin. Scale bar=100 *μ*m.

Evaluation of antitumor activity of H_2_Mab-19 in BT-474 xenografts. (A) BT-474 xenografts on day 18. (B) Resected tumors of BT-474 xenografts (day 18). (C) Body weights of the mice with the BT-474 xenografts. n.s., not significant. Scale bar=1 cm.

Immunohistochemical analyses and hematoxylin & eosin (HE) staining of resected tissues in BT-474 xenografts. Sections were incubated with H_2_Mab-19 (10 *μ*g/ml) or H_2_Mab-77 (10 *μ*g/ml). Sections were then counterstained with hematoxylin. HE staining was also performed. Scale bar=100 *μ*m.

Evaluation of antitumor activity of H_2_Mab-19 in HSC-2 xenografts. (A) HSC-2 xenografts on day 20. (B) Resected tumors of HSC-2 xenografts (day 20). (C) Body weights of mice with HSC-2 xenografts. n.s., not significant. Scale bar=1 cm.

Immunohistochemical analyses and hematoxylin & eosin (HE) staining of resected tissues in HSC-2 xenografts. Sections were incubated with H_2_Mab-19 (10 *μ*g/ml) or H_2_Mab-77 (10 *μ*g/ml). Then, sections were counterstained with hematoxylin. HE staining was also performed. Scale bar=100 *μ*m.

Evaluation of antitumor activity of H_2_Mab-19 in SAS xenografts. (A) SAS xenografts on day 20. (B) Resected tumors of SAS xenografts (day 20). (C) Body weights of mice with the SAS xenografts. n.s., not significant. Scale bar=1 cm.

Immunohistochemical analyses and hematoxylin & eosin (HE) staining of resected tissues in SAS xenografts. Sections were incubated with H_2_Mab-19 (10 *μ*g/ml) or H_2_Mab-77 (10 *μ*g/ml). Then, sections were counterstained with hematoxylin. HE staining was also performed. Scale bar=100 *μ*m.

## Data Availability

The datasets used and/or analyzed during the study are available from the corresponding author on reasonable request.
