# Supplementary material for: H2Mab-19, an anti-human epidermal growth factor receptor 2 monoclonal antibody exerts antitumor activity in mouse oral cancer xenografts
Source: Exp Ther Med. 2020 May 18;20(2):846–53. doi: 10.3892/etm.2020.8765 (PMC7388441; doi:10.3892/etm.2020.8765)

Figure S1. Immunohistochemical analyses of paraffin sections of breast cancers using H_2Mab-19 and H_2Mab-77. Sections of breast cancers were incubated with H_2Mab-19 (10 μg/ml) or H_2Mab-77 (1 μg/ml) Sections were counterstained with hematoxylin. Scale bar=100 μm.

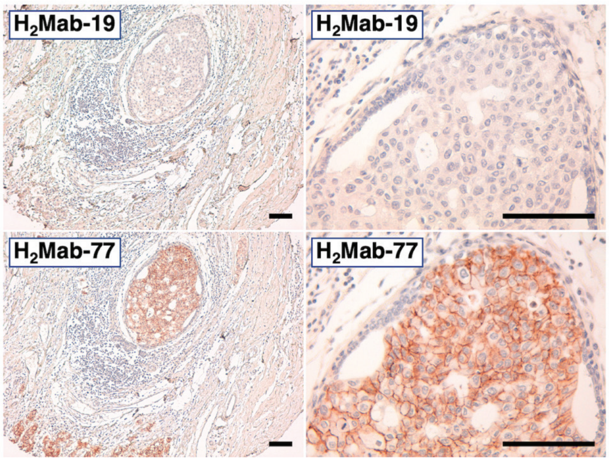

Figure S2. Immunohistochemical analyses of frozen sections of breast cancers using H_2Mab-19 or H_2Mab-77. Sections of breast cancers were incubated with H_2Mab-19 (1 μg/ml) or H_2Mab-77 (1 μg/ml). Sections were then counterstained with hematoxylin. Scale bar=100 μm.

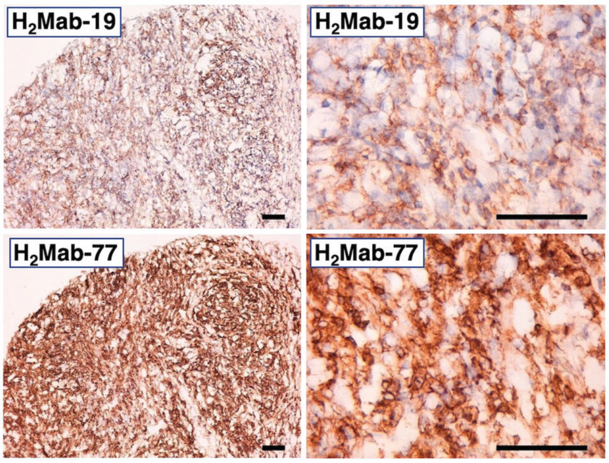

Figure S3. Evaluation of antitumor activity of H_2Mab-19 in BT-474 xenografts. (A) BT-474 xenografts on day 18. (B) Resected tumors of BT-474 xenografts (day 18). (C) Body weights of the mice with the BT-474 xenografts. n.s., not significant. Scale bar=1 cm.

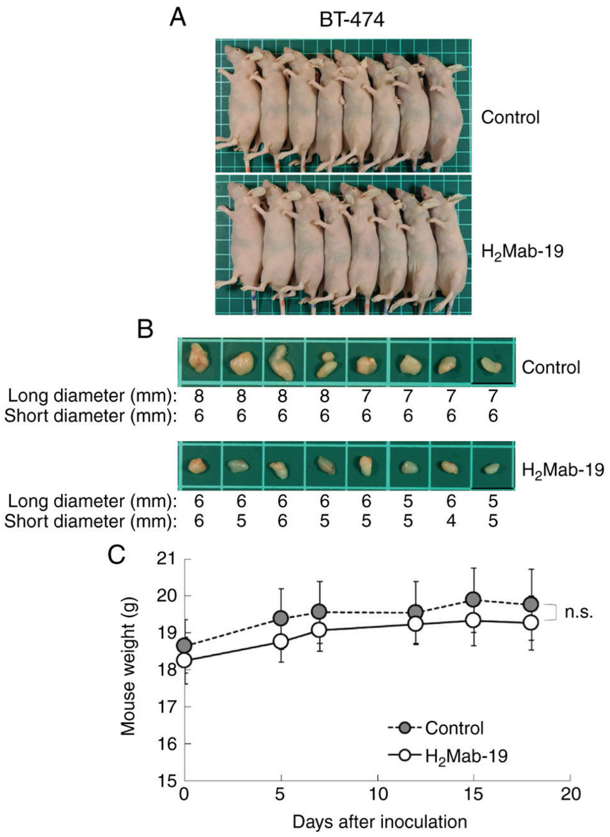

Figure S4. Immunohistochemical analyses and hematoxylin & eosin (HE) staining of resected tissues in BT-474 xenografts. Sections were incubated with H_2Mab-19 (10 μg/ml) or H_2Mab-77 (10 μg/ml). Sections were then counterstained with hematoxylin. HE staining was also performed. Scale bar=100 μm.

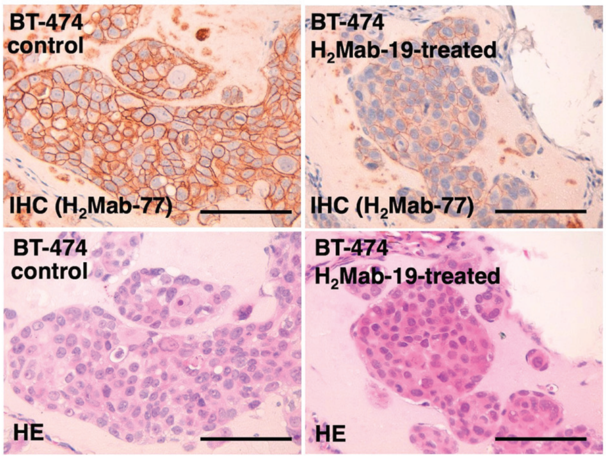

Figure S5. Evaluation of antitumor activity of H_2Mab-19 in HSC-2 xenografts. (A) HSC-2 xenografts on day 20. (B) Resected tumors of HSC-2 xenografts (day 20). (C) Body weights of mice with HSC-2 xenografts. n.s., not significant. Scale bar=1 cm.

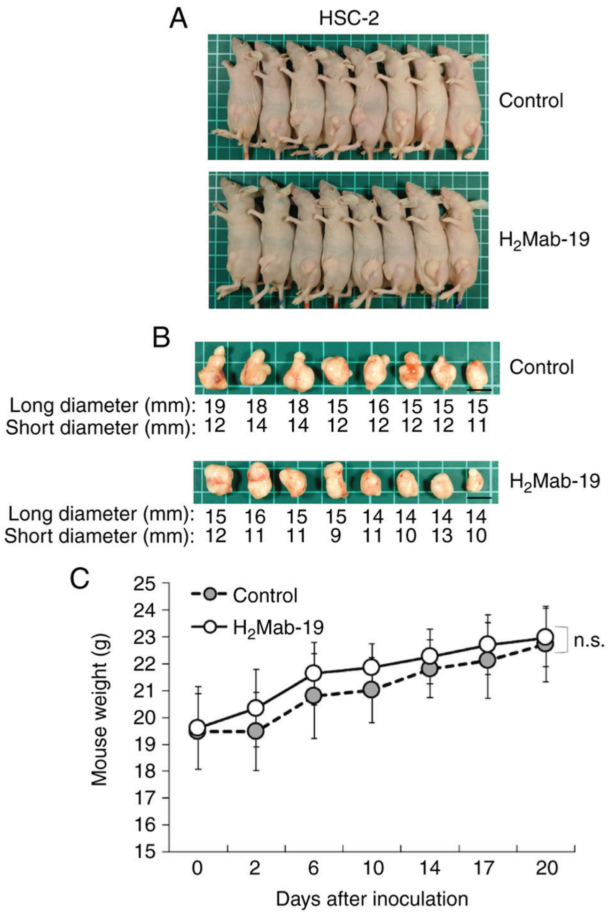

Figure S6. Immunohistochemical analyses and hematoxylin & eosin (HE) staining of resected tissues in HSC-2 xenografts. Sections were incubated with H_2Mab-19 (10 μg/ml) or H_2Mab-77 (10 μg/ml). Then, sections were counterstained with hematoxylin. HE staining was also performed. Scale bar=100 μm.

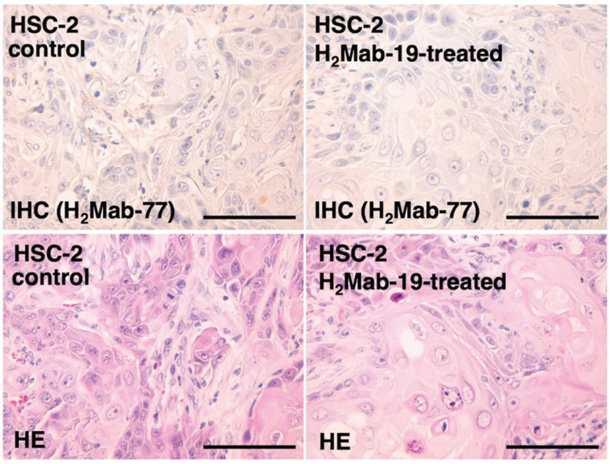

Figure S7. Evaluation of antitumor activity of H_2Mab-19 in SAS xenografts. (A) SAS xenografts on day 20. (B) Resected tumors of SAS xenografts (day 20). (C) Body weights of mice with the SAS xenografts. n.s., not significant. Scale bar=1 cm.

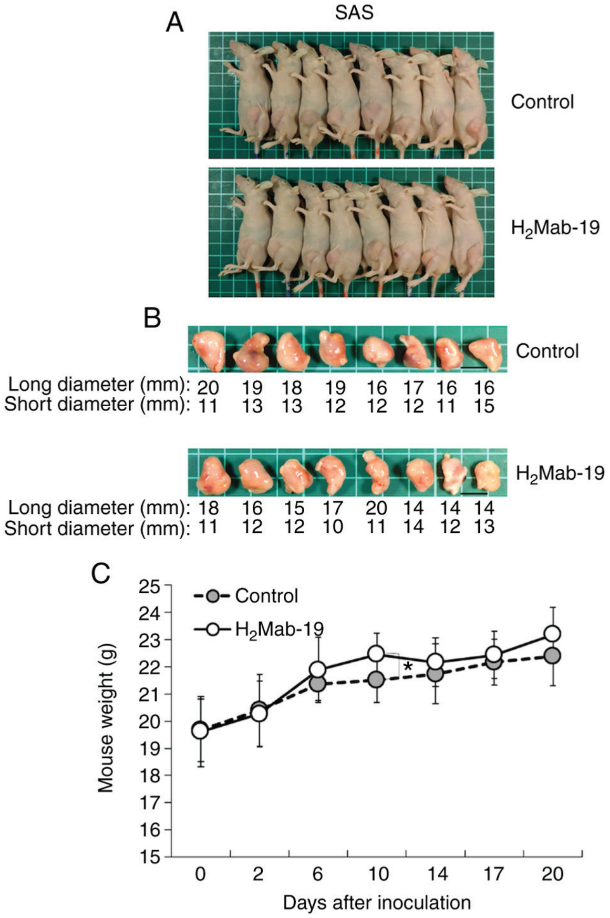

Figure S8. Immunohistochemical analyses and hematoxylin & eosin (HE) staining of resected tissues in SAS xenografts. Sections were incubated with H_2Mab-19 (10 μg/ml) or H_2Mab-77 (10 μg/ml). Then, sections were counterstained with hematoxylin. HE staining was also performed. Scale bar=100 μm.

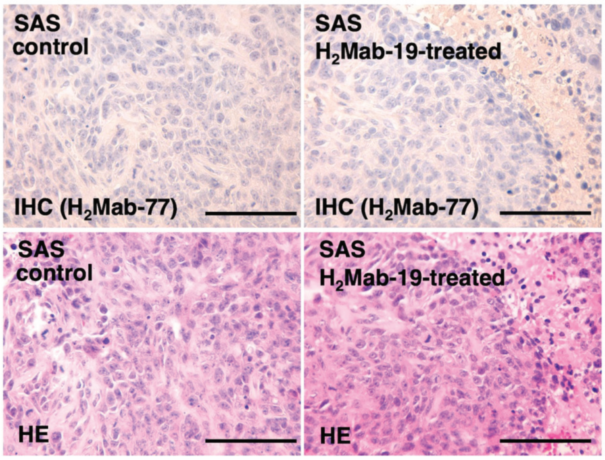

Supplement: Immunohistochemical analyses of paraffin sections of breast cancers using H2Mab-19 and H2Mab-77. Sections of breast cancers were incubated with H2Mab-19 (10 μg/ml) or H2Mab-77 (1 μg/ml) Sections were counterstained with hematoxylin. Scale bar=100 μm. [file Supplementary_Data.pdf]
